# Prognostic Factors Associated with 5-Year Overall Survival in Cervical Cancer Patients Treated with Radical Hysterectomy Followed by Adjuvant Concurrent Chemoradiation Therapy at a Tertiary Care Center in Eastern Europe

**DOI:** 10.3390/diagnostics11030570

**Published:** 2021-03-22

**Authors:** Mihai Stanca, Mihai Emil Căpîlna

**Affiliations:** First Obstetrics and Gynecology Clinic, University of Medicine, Pharmacy, Science and Technology “G.E. Palade” of Târgu Mureș, 540142 Târgu Mureș, Romania; mcapilna@gmail.com

**Keywords:** cervical cancer, radical hysterectomy, prognostic factors, overall survival, chemoradiotherapy

## Abstract

Background: This retrospective observational study aims to assess the 5-year overall survival and the prognostic significance of risk factors of patients who underwent radical hysterectomy followed by adjuvant concurrent chemoradiation therapy (CCRT) for FIGO stage IB1-IIB cervical cancer in a tertiary care center in Eastern Europe. Methods: From January 2010 to February 2019, 222 patients with stage IB1-IIB cervical cancer were treated with radical hysterectomy followed by adjuvant CCRT in our institution. The baseline information consisting of demographic and clinicopathologic data, treatment choices, recurrences, and outcome information was collected and examined. The survival rates were illustrated using Kaplan–Meier curves and prognosis analyses were accomplished using Cox multivariate analyses. Results: The 222 participants had a mean age of 51.2 years (28–76). The median follow-up time was 65.5 months (3–128). Tumor characteristics revealed FIGO stage (IB1 2.3%, IB2 35.1%, IB3 16.7%, IIA1 9%, IIA2 8.6%, IIB 28.4%) and the most encountered histologic cell type was squamous cell carcinoma (80.06%) followed by adenocarcinoma (11.3%). At the time of examination, 157 patients (70.07%) were alive, of which 135 (61%) were alive free of disease and 22 (9%) were alive with disease. The multivariate Cox regression analysis acknowledged stage IIB, parametrial involvement, and the presence of lymph node metastases as independent prognostic risk factors, significantly worsening the oncologic outcomes influencing the survival with a *P*-value of 0.076, 0.0001, and 0.008, respectively. The 5-year overall survival was 69.9%. Conclusions: Altogether, the study enhances the significance of prognostic risk factors on the 5-year overall survival of patients who underwent radical hysterectomy followed by adjuvant CCRT for FIGO stages IB1-IIB cervical cancer, allowing comparisons with other regions.

## 1. Introduction

Worldwide, every 2 min a woman dies from cervical cancer [1]. Concerning women, cervical cancer ranks fourth in both frequency and mortality, with approximately 604,000 new cases and 342,000 deaths in 2020 [2]. The 5-year overall survival of women diagnosed with cervical cancer in Europe is 66%, with a particularly lower percentage of those from Eastern Europe (<55%) [3].

Radical hysterectomy with pelvic lymph node dissection is commonly acknowledged as a standard of care for patients with early-stage disease (stage IA2-IIA) [4]. While conclusive chemoradiotherapy is deemed as a usual treatment for FIGO stage IIB cervical cancer, the role of surgery remains debatable [5]. The radical hysterectomy has to be followed by adjuvant chemoradiotherapy to improve local control in patients with pathological risk factors.

Cervical cancer experienced a radical change in the treatment procedure upon the discovery that patients who received radical hysterectomy followed by adjuvant concurrent chemoradiation therapy (CCRT) had longer surviving rates than those who have received adjuvant radiotherapy or no adjuvant therapy at all [6,7,8].

The current study assesses the influence of risk factors on the 5-year overall survival in stage IB1-IIB cervical cancer patients who underwent radical hysterectomy followed by adjuvant CCRT in a 9-year follow-up study at a tertiary care center in Eastern Europe, allowing comparisons with other regions.

The need for this research lies in the fact that Eastern Europe has faced an increasingly alarming number of new cases and premature deaths caused by cervical cancer both due to poor screening and incorrectly applied treatments. In Eastern Europe, there are 14.5 new cases and 6.1 deaths per 100,000 inhabitants versus 7 new cases and 2 deaths per 100,000 inhabitants in Western Europe [2]. Accordingly, Romania has the highest standardized rates of incidence and mortality from cervical cancer in Europe [9].

## 2. Materials and Methods

### 2.1. Patient Population

The current study evaluates the medical records of 222 out of 430 patients who underwent type C2 Querleu–Morrow radical hysterectomy with pelvic lymph node (LN) dissection and adjuvant (CCRT) for cervical cancer stage IB to IIB with at least one or more intermediate-risk or high-risk factors, respectively, at the First Obstetrics and Gynecology Clinic of Târgu Mureș, Romania, from January 2010 to February 2019.

The clinical staging was performed according to FIGO 2018 guidelines [4]. The study was conducted under the approval of the Institutional Review Board (IRB) of our Institute (ethical approval code: 34535/13.12.2019) and written informed consent was obtained from all subjects.

The baseline information consisting of demographic and clinicopathologic data was attained from patients’ medical records. These data included age, urban or rural provenience, FIGO stage, tumor size, histologic subtype, tumor differentiation grade, depth of cervical stromal invasion, lymphovascular space invasion, parametrial involvement, resection margin status, LN status, the presence of recurrence, date of death, and patient condition at last follow-up.

Patients with positive para-aortic lymph-nodes or who received chemotherapy (CT), radiotherapy (RT) or CCRT before the radical hysterectomy and patients who received postoperative adjuvant CT or RT alone were excluded. Furthermore, patients whose follow-up had been lost were left out of the study.

### 2.2. Treatments

All patients included in the present study underwent radical hysterectomy with pelvic LN dissection and postoperative adjuvant CCRT. The adjuvant CCRT dosages differed due to the postoperative pathology reports and the patients’ wishes.

The external beam of RT aimed to include the entire pelvis with fractionation of 1.8–2.0 Gy tumor dose daily with five fractions per week with a medium intake dosage of 48.5 Gy (45–50 Gy). In terms of chemotherapy, patients received weekly cisplatin during RT or 5-fluorouracil-cisplatin (FP) combination.

### 2.3. Follow-Up

After concluding the treatment, consultations were scheduled every 3 months in the first 2 years, every 6 months for the next 3 years, then annually until October 31, 2020.

A complete physical, gynecological, laboratory, and imaging examination was performed at each medical follow-up visit.

The major endpoints of the study were the 5-year overall survival (OS), which was defined as the time from surgery to the most recent follow-up or death, and the influence of different prognostic factors (intermediate and high-risk factors) on the overall survival.

The death caused by cervical cancer was recorded by telephone or via letters received from the patients’ relatives.

### 2.4. Statistical Analysis

Differences between categorical variables were determined using the X^2^ test. Differences between numerical variables were determined by t-test. *P* < 0.05 was considered statistically significant. The survival curves were evaluated using the Kaplan–Meier method. Survival rates were compared using the Log-rank test, and risk factors for the prognosis were assessed by Cox regression analysis.

A Cox proportional hazards model with 95% confidence intervals (95% CI) was used to carry out multivariable analysis of various factors affecting the overall survival. IBM SPSS software 23.0 was used for statistical analyses.

## 3. Results

### 3.1. Patient Characteristics

Out of the 430 patients undergoing radical hysterectomy with pelvic LN dissection between January 2010 and February 2019, only 222 met the inclusion criteria in the current study. Therefore, all 222 patients (100%) received adjuvant CCRT after surgery and had at least one high or intermediate postoperative pathological risk factor.

The mean age of the participants was 51.2 years (28–76). Tumor characteristics revealed FIGO stage (IB1 2.3%, IB2 35.1%, IB3 16.7%, IIA1 9%, IIA2 8.6%, IIB 28.4%), the most encountered histologic cell type was squamous cell carcinoma (80.06%) followed by adenocarcinoma (11.3%). A total of 42.3% of the patients had parametrial invasion, the LN metastases were approximately equal and only 23% had positive surgical margins. The detailed patient characteristics and clinicopathological data are summarized in Table 1.

Out of the 63 patients with preoperative clinical stage IIB, 67% had histopathologically confirmed parametrial invasion, and the remaining 33% had, in fact, a pseudo clinical stage IIB.

### 3.2. Survival Analysis

**Figure 1:**
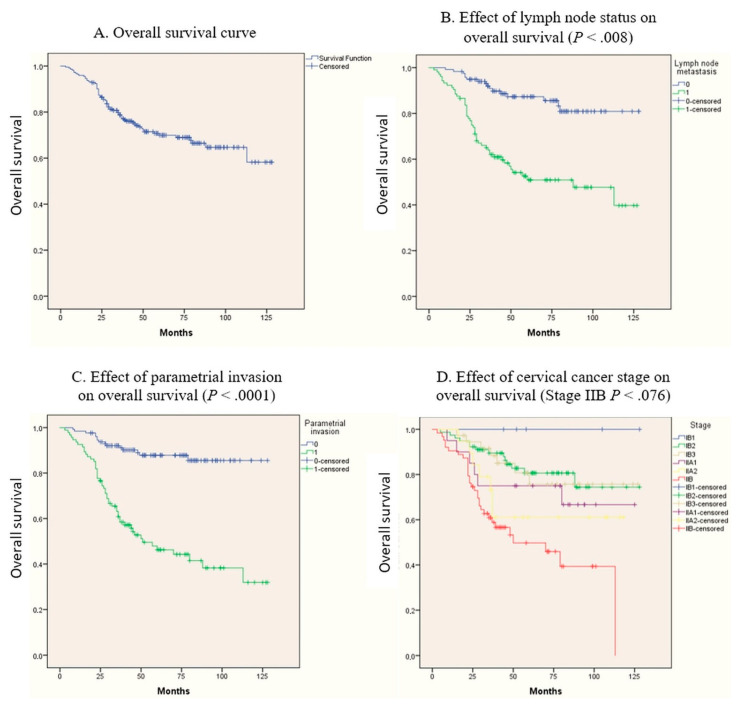
The most representative Kaplan–Meier curves.

Patient follow-up lasted until 31 October 2020, with a follow-up average of 65.5 months (3–128). At the analysis time, out of the 222 patients, 157 (70.07%) were alive, out of which 135 (61%) were alive free of disease and 22 (9%) were alive with disease. The 5-year overall survival was 69.9%. Major intraoperative bleeding was avoided [10]; no deaths were caused by postoperative complications or CCRT treatments. Details are available in Table 1 and the most representative Kaplan–Meier curves are presented in Figure 1.

The univariate analysis shows that the survival prognosis was directly influenced by tumor stage IB2, IIB as well as parametrial impairment, and the presence of positive lymph nodes, all with a *P*-value <.05% as seen in Table 1. As expected, the multivariate Cox regression analysis acknowledged the stage IIB, parametrial involvement, and the lymph node metastases as independent prognostic risk factors, influencing the survival with a *P*-value of 0.076, 0.0001 and 0.008 respectively (Table 2).

Because out of the 22 patients alive with disease most did not perform the medical examinations at the recommended intervals, the authors were unable to record the exact date of recurrence. To avoid bias in the data analysis, no data were recorded in this aspect. Nevertheless, in this setting of patients, it was observed that the clinical-stage FIGO IIB is an independent prognostic factor for disease recurrence (*P* < 0.02) (Table 1.).

## 4. Discussion

Radical hysterectomy with pelvic lymph node dissection is commonly acknowledged as a standard of care for patients with early-stage disease (stage IA2-IIA) [4]. While conclusive chemoradiotherapy is deemed as the treatment of choice for FIGO stage IIB cervical cancer, the role of surgery remains debatable [5]. The low pre- and postinterventional accordance of parametrial involvement suggest that more than half of the patients with FIGO stage IIB have “pseudo” stage IIB disease, which could be dealt with a radical hysterectomy followed by adjuvant CCRT but not definitive neoadjuvant radio or chemoradiotherapy alone [5,11,12]. However, in the current study, out of the 63 patients with preoperative clinical stage IIB, 67% had histopathologically confirmed parametrial invasion, with only 33% having an overvalued clinical stage IIB.

The radical surgery has to be followed by adjuvant CCRT to improve local control in patients with pathological risk factors. According to the National Comprehensive Cancer Network [13] and ESMO Clinical Practice Guidelines [14], some elements have been identified as high-risk factors, including microscopic parametrial involvement, LN metastasis, and positive or close surgical margins. For this category of patients, adjuvant CCRT is advised [8,15]. The parametrial invasion and the existence of positive LN influenced the overall survival of the patients included in the current study while the presence of positive surgical margins had only a minor role (Table 1).

Corresponding to Sedlis standards [16], the intermediate-risk factors consist of the presence of lymphovascular space invasion, tumors greater than ≥4 cm in size, and deep cervical stromal invasion. The presence of these factors does not substantially increase the recurrence rate alone but, when merged, the risk of recurrence is augmented to 15–20% [14]. In the case of two or more being mutually present, adjuvant CCRT should be suggested as it has revealed a statistical advantage in terms of progression-free survival [8]. In the current study, these elements slightly influenced overall survival.

Potential risk factors may not be restricted to the Sedlis standards. The prognostic significance of tumor differentiation grade has been debated, and historically, tumor grade has not been included in treatment decision-making for cervical cancer according to several studies performed a few decades ago [17,18]. However, there are recent studies that shed light on the importance of this aspect. A higher tumor grade is linked to diminished survival rates [19], though considering the tumor differentiation grade into the treatment procedure has not been yet assessed and additional study is necessary. In the current study, the poorly-differentiated tumor slightly modified the 5-year overall survival (Table 1.).

Furthermore, there are disagreements as to whether the histological type is a self-determining prognostic factor for survival or not. Many studies have shown that adenocarcinoma is responsible for a worse prognosis compared with squamous cell carcinoma [14,20]. The negative effect of adenocarcinoma on the 5-year overall survival was also noticed in the current study, as patients with adenocarcinoma had a 68.5% survival rate versus 78.6% of those with squamous cell carcinoma.

Generally, most patients with early-stage cervical cancer undergo radical surgery [8]. The treatment of cervical cancer experienced a radical change in approach upon the discovery that patients who received radical hysterectomy followed by adjuvant CCRT had longer surviving rates than those who have received adjuvant radiotherapy alone [6,7,8]. According to the NCCN guidelines [13], patients who have high-risk factors at the final histopathological examination should uniformly receive adjuvant CCRT as this is the treatment of choice to reduce recurrences of the disease and increase overall survival [21,22].

In most cases, patients do not have a single risk factor, but several. For instance, parametrial invasion along with LN metastases dramatically influences survival [23].

The present study identified a major survival difference in patients with high-risk factors with a higher probability of early death. Therefore, the multivariate analysis identified the clinical FIGO stage IIB, parametrial involvement, and lymph node metastases as independent prognostic risk factors influencing survival with *P* values of 0.076, 0.0001, and 0.008, correspondingly.

In a comparable study, similar to the results obtained by the authors, Lim et al. [22] found that patients who had one or more high-risk factors had higher death rates.

The presence of LN metastases, the parametrial involvement, and the positive resection margins are linked with an unfavorable prognosis in patients with early-stage cervical cancer [24]. The LN metastases are the most accurate predictor of survival in women with cervical cancer who underwent a radical hysterectomy [8,25,26].

Although positive resection margins are considered among the high-risk factors, the present study did not show a significant association between their existence and death. This may reside in the effectiveness of adjuvant CCRT.

The 5-year survival is also influenced by histology results, tumor size, deep stromal invasion, lymphovascular space invasion, and certain HPV strains [8,16,27,28]. However, no effective statistical associations related to these elements were found in the present study nor regarding the tumor differentiation grade.

Yan et al. [29] also demonstrated an improved prognosis for the survival of IB1-IIB stage cervical cancer patients with high-risk factors or more than two intermediate-risk factors undergoing adjuvant CCRT therapy. However, patients with intermediate-risk factors do not benefit from a standardized treatment regimen, which requires therapy management under the experience of clinicians [29].

The strengths of this study are a considerable number of patients (*n* = 222) and a long study period (9 years). Moreover, all patients benefited from a uniform therapeutic scheme, granted at a single institution. These aspects better mirror the impact of different risk factors on the prognosis. However, the findings from this study only intensify the negative effects of high-risk factors and the moderate effects of intermediate-risk factors on the prognosis of cervical cancer patients. The main outcome demonstrates a 5-year overall survival of 69.9% which is increasingly different from the data provided by the latest research in the field which reveals a 5-year overall survival of less than 55% in Eastern European countries [3]. This underlines the effectiveness of a multidisciplinary team approach and of the adjuvant CCRT. Furthermore, the undeniable importance of performing this kind of procedure in centers with experience in ultraradical surgery is not to be overlooked [30].

As this is a retrospective observational study, the authors recognize several limitations. Although these studies have a poorer level of evidence in comparison with prospective ones and are subject to misperceptions, not being able to reveal causation, only associations, they can detect potential risk factors [31]. A major limitation of the study would have to be the lack of data interpretation regarding the 22 patients with recurrent disease. Most did not show up at the appointed medical visits. To avoid bias in the data analysis, no major data were recorded for this aspect.

## 5. Conclusions

The present study shows the prognostic value of risk factors on the 5-year overall survival in a setting of patients from Eastern Europe who underwent radical hysterectomy followed by adjuvant CCRT for FIGO stages IB1-IIB cervical cancer, allowing comparisons with other regions. The multivariate Cox analysis revealed that the clinical-stage FIGO IIB, parametrial invasion, and the presence of LN metastases were associated with significantly worse oncologic outcomes, interpreted into lower overall survival. Altogether, the information provided by this study adds more evidence regarding the value of prognostic risk factors in patients with cervical cancer.

## Figures and Tables

**Table 1:** Demographic and clinical characteristics of eligible participants; the univariate 5-year overall survival and the recurrence analyses.

	Number (%) or Median (Range)	Overall Survival				Recurrences	
		5-Year Survival Rate	95% CI	Mean Survival (Months)	*P* Value	Number	*P* Value
No. of Patients	222					22	
Age (Years)	51.2 (28–76)						
30–40		62.7%	61.9–63.5	90.5	0.740		
41–50		66.4%	65.6–67.1	95.9	0.940		
51–60		67.8%	67.1–68.4	93	0.909		
61–70		65.3%	64.3–66.2	90.4	0.876		
**Provenance**							
Urban	92 (41.4%)					12	
Rural	130 (58.6%)					10	
**Stage (FIGO 2018)**							
IB1	5 (2.3%)	100%			0.350		
IB2	78 (35.1%)	80%	79.5–80.5		0.009	4	0.060
IB3	37 (16.7%)	75.7%	74.8–76.5		0.131	4	0.518
IIA1	20 (9.0%)	66.7%	65.5–67.8		0.802	2	0.615
IIA2	19 (8.6%)	61.1%	59.94–62.2		0.585	1	0.415
IIB	63 (28.4%)	39.3%	38.4–40.2		0.001	11	0.020
**Tumor size**					0.603		.475
<4 cm	167 (75.2%)	70%	69.6–70.3			16	
≥4 cm	55 (24.8%)	68.3%	67.5–69			6	
**Histology**							
Squamous Cell Carcinoma	179 (80.6%)	78.6%	78.2–78.9	94.6	0.856	18	0.572
Adenocarcinoma	25 (11.3%)	68.5%	67.5–69.5	96.6	0.896	2	0.537
Other	18 (8.1%)	61.9%	60.6–63	88.4	0.675	2	0.554
**Tumor Differentiation Grade**							
Grade 1 (Well-Differentiated)	20 (9%)	83.8%	82.9–84.6	92.3	0.331	1	0.615
Grade 2 (Moderately-Differentiated)	82 (36.9%)	71.6%	71–72.1	96.8	0.931	11	0.135
Grade 3 (Poorly-Differentiated)	120 (54.1%)	69.7%	69.2–70.1	91.9	0.615	9	0.141
**Depth of Cervical Stromal Invasion**							
Inner 1/3	14 (6.3%)	73%	71.6–74.4	87.3	0.709	1	0.586
Middle 1/3	26 (11.7%)	66.8%	65.5–66.9	98.5	0.749	1	0.238
Outer 1/3	182 (82.0%)	75.5%	75.1–75.8	93.3	0.398	20	0.200
**Lymphovascular Space Invasion**					0.762		0.207
Positive	171 (77.0%)	77.4%	77–77.7	94.2		19	
Negative	51 (23.0%)	68.4%	67.7–69	94.7		3	
**Parametrial Involvement**					0.0001		0.463
Positive	94 (42.3%)	46.3%	45.7–46.8	69.9		10	
Negative	128 (57.7%)	87.8%	87.4–88.1	114.7		12	
**Resection Margin Status**					0.801		0.109
Positive	52 (23%)	69.6%	68.9–70.5	94.3		8	
Negative	170 (77%)	70%	69.6–70.3	94.1		14	
**Pelvic Lymph Nodes Metastases**					0.0001		0.162
Positive	104 (47%)	58.3%	57.8–58.8	76.1		13	
Negative	118(53%)	87.3%	86.9–87.6	112.3		9	
**Adjuvant Treatment**							
CCRT	222 (100%)						
Median Follow-Up Duration (Months)	65.5 (3–128)						
**Status**							
Alive	157 (70.07%)	69.9%	69.5–70.2	94.7			
Alive Free of Disease	135 (61%)						
Alive with Disease	22 (9%)						
Deceased	65 (29.3%)						

**Table 2:** Multivariate Cox proportional hazard regression analysis of predictors of overall survival.

Variables	B	SE	Wald	*P*-Value	HR	95.0% CI for OR	
						Lower	Upper
**IIB**	0.535	0.301	3.145	.076	1.707	0.945	3.082
**Parametrial Invasion**	1.200	0.333	12.979	.0001	3.322	1.729	6.383
**Lymph Node Metastasis**	0.838	0.314	7.140	.008	2.313	1.250	4.277

B, regression coefficient; SE, standard error; Wald, X^2^ value equal to B2 divided by its standard error; HR, hazard ratio; CI, confidence interval; OR, odds ratio.

## Data Availability

We provide our data for the reproducibility of this study in other centers if such is requested.
